# Retinal Microvasculature and Neural Changes and Dietary Patterns in an Older Population in Southern Italy

**DOI:** 10.3390/ijerph20065108

**Published:** 2023-03-14

**Authors:** Rossella Tatoli, Luisa Lampignano, Rossella Donghia, Alfredo Niro, Fabio Castellana, Ilaria Bortone, Roberta Zupo, Sarah Tirelli, Madia Lozupone, Francesco Panza, Giovanni Alessio, Francesco Boscia, Giancarlo Sborgia, on behalf of the Eye Clinic Research Group, Rodolfo Sardone

**Affiliations:** 1 National Institute of Gastroenterology—IRCCS “Saverio de Bellis”, Research Hospital, 70013 Bari, Italy; 2 Eye Clinic, Hospital “SS. Annunziata”, ASL Taranto, 74100 Taranto, Italy; 3 Department of Translational Biomedicine and Neuroscience “DiBraiN”, University of Bari Aldo Moro, 70121 Bari, Italy

**Keywords:** retina, older population, nutrition, OCT, diet

## Abstract

Background: Like other parts of the body, the retina and its neurovascular system are also affected by age-related changes. The rising age of populations worldwide makes it important to study the pathologies related to age and their potential risk factors, such as diet and eating habits. The aim of this study was to investigate the predictive power of food groups versus retinal features among noninstitutionalized older adults from Southern Italy using a machine learning approach. Methods: We recruited 530 subjects, with a mean age of 74 years, who were drawn from the large population of the Salus in Apulia Study. In the present cross-sectional study, eating habits were assessed with a validated food frequency questionnaire. For the visual assessment, a complete ophthalmic examination and optical coherence tomography-angiography analyses were performed. Results: The analyses identified 13 out of the 28 food groups as predictors of all our retinal variables: grains, legumes, olives-vegetable oil, fruiting vegetables, other vegetables, fruits, sweets, fish, dairy, low-fat dairy, red meat, white meat, and processed meat. Conclusions: Eating habits and food consumption may be important risk factors for age-related retinal changes. A diet that provides the optimal intake of specific nutrients with antioxidant and anti-inflammatory powers, including carotenoids and omega-3 fatty acids, could have beneficial effects.

## 1. Introduction

In our aging society, age-related diseases are an issue of great public interest. Age is a real risk factor for several chronic inflammatory diseases, altering the structure and/or function of organs and tissues [1]. Worldwide, the increasing number of subjects over 60 years of age has led to progressively growing interest in age-related clinical disorders and diseases. It is now essential to investigate their principal modifiable risk factors to identify new and possible strategies to prevent or regress retinal damage. The retina and its cellular and microvascular components are among the districts affected by these age-related changes that can impair their function, leading to gradual or sudden visual loss [2].

The retinal tissue plays a specific role in processing visual information. It has a complex structure composed of pigment epithelium, photoreceptor cells (rods and cones), and more than 60 distinct types of neuronal cells and their fibers [2]. The pigment epithelium is rich in melanin cells, highly pigmented, and is called retinal pigment epithelium (RPE). This type of cell plays numerous crucial roles in the health and normal functioning of the retina, including the delivery of nutrients to the photoreceptor cells and the transportation of ions, metabolites, and fluids to the blood-retinal barrier. At the center of the retina is the macula, a highly pigmented area containing the central fovea, affecting the clarity of vision [3]. The correct functioning of the RPE, the macula, and the central fovea are significant for the health of the retina, and any degeneration can have serious consequences on the visual process. Aging physiologically determines some cellular changes, such as reactive oxygen species (ROS) production, oxidative stress, metabolic dysfunction, and inflammatory responses that can potentially disrupt tissue homeostasis [4,5,6]. This leads to the activation of an adaptation mechanism known as cellular senescence, a cellular response induced by different types of stress that accompanies normal aging [5]. Disproportionate changes induced by cellular senescence, or a persistent accumulation of senescent cells due to chronic stress, exacerbate the adverse effects of aging and can lead to some clinical illnesses and different chronic disorders, including age-related macular degeneration (AMD) [7,8,9]. AMD is a complex medical condition that damages a part of the retina and the macula and compromises the central vision and the ability to see fine details. It is a socially debilitating condition that reduces the quality of life because it leads to loss of balance, mobility and independence issues, depression, and social isolation [10].

AMD is an age-related disease that is currently the first cause of visual impairment and central blindness in the Western population over 65 years of age. Globally, it affects 30–50 million individuals and is expected to increase ten-fold by 2040 [11]. In Italy, AMD affects one million people, and it is estimated that this number will increase with further population aging [12]. The National Eye Institute sponsored two clinical trials, The Age-Related Eye Disease Study (AREDS) and AREDS2, to learn more about AMD, its natural history, and risk factors [13]. The characteristic “drusen” of AMD is caused by hyaline deposits accumulating between the RPE and Bruch’s membrane. The size of the drusen is different in the various disease stages. In the early stages, a small drusen (63 to 124 μm in diameter) is present with hyper/hypopigmentations of the retinal epithelium [14]. In this stage, mild symptoms may or may not be present, but if they are, they will include blurred vision and impaired dark adaptation [15]. In the advanced forms, the drusen size increases (by 125 μm in diameter), and moderate to severe visual loss is present [13,14]. Late AMD can be distinguished as the nonexudative dry AMD (Geographic Atrophy, GA) and the exudative wet form (Neovascular AMD) [16].

Moreover, findings in the retina provide a noninvasive way to investigate the systemic health of the human body. Particularly, subjects with an increased risk of liver fibrosis [17] or hypertension [18] had thinner neuroretinal layers, according to high-resolution retinal scan images taken with optical coherence tomography (OCT) technology. OCT-angiography (OCT-A) allows for the study of retinal health and its microvascular network; this innovative version uses OCT technology to obtain a noninvasive depth-resolved visualization of the retinal microvasculature [19,20]. 

The main risk factors related to the development and progression of age-related retinal changes include gender, race, genetics, environmental, lifestyle, and dietary factors. For example, the Caucasian female population over the age of 65 is at greater risk [21,22]; environmental and lifestyle factors include light exposure and smoking [23,24]. Chakravarthy and colleagues reported that among older Europeans, the risk of AMD in smokers was 5 and 2.5 times higher for the dry and neurovascular forms, respectively [25]. Cigarette smoking doubles the risk of developing this condition and causes retinal damage through pro-oxidative and proinflammatory processes [25].

In these processes, a clear role could be played by inflammation and oxidative stress [26]. In fact, another risk factor is a diet with a poor content of vitamins A, C, and E and zinc, lutein, zeaxanthin, and omega-3 fatty acids such as DHA [27]. Currently, no curative treatment has been identified for AMD. Antivascular-endothelial growth factor (VEGF) drugs are used to treat the wet form, while no therapy is available to slow the progression of dry AMD. In the last decades, several studies have been focused on researching preventive measures and strategies to slow the degeneration related to this condition. AREDS 1 and 2 mainly evaluated the role of some nutrients and nutritional supplementation in preventing and reversing these eye diseases. A diet based on vegetables, fruits, unrefined products, fish, and extra virgin olive oil could be a valuable tool due to its protective properties against various noncommunicable diseases [28]. Studies demonstrated how adopting proper eating habits also reduces the risk of running into the vascular complications of diabetes, including diabetic retinopathy (DR) [29]. Oxidative stress and inflammation have also been shown to play a key role in DR pathophysiology [30] and antioxidants in its prevention and treatment. The therapeutic role of polyphenols and polyunsaturated fatty acids (PUFA) and the preventive role of vitamin C, vitamin E, lutein, and zeaxanthin have been hypothesized [31,32,33].

More attention should be paid to the dietary and nutritional aspects regarding the prevention and/or treatment of age-related visual degeneration. The present study aimed to investigate the relationship between retinal features and eating habits among noninstitutionalized older adults from Southern Italy using a machine learning approach. This is a new approach that has not been widely used in previous studies on the retina and its components [34].

## 2. Materials and Methods

### 2.1. Study Design and Population

This cross-sectional population-based study involved 530 subjects aged over 64. This was a subsample drawn from the “Salus in Apulia Study”, a public health initiative promoted by the Italian Ministry of Health and Apulia Regional Government and conducted at IRCCS “S. de Bellis” Research Hospital in Castellana Grotte, Southern Italy. In this study, 4537 individuals were enrolled from 2014 to 2019 in Castellana Grotte. This selection of study participants allowed us to utilize past individual data generated from other investigations. The sample is representative of the entire population of older people (age > 65 years) from Castellana Grotte in 2014, as described elsewhere [35]. In this study, we analyzed the data from those subjects who had both undergone nutritional and ophthalmological assessments. The IRB of the head institution, the National Institute of Gastroenterology and Research Hospital “S. de Bellis” in Castellana Grotte, Italy, approved the study. The study was conducted according to the Helsinki Declaration of 1975 and adhered to the “Standards for Reporting Diagnostic Accuracy Studies” (STARD) guidelines (http://www.stard-statement.org/ accessed on 22 December 2022). The manuscript was organized according to the “Strengthening the Reporting of Observational Studies in Epidemiology-Nutritional Epidemiology” (STROBE-nut) guidelines (https://www.strobe-nut.org/ accessed on 22 December 2022). For the present study, the participants were subject to nutritional and visual assessments. All of them signed an informed consent form before examination. 

### 2.2. Clinical and Lifestyle Assessment

The anthropometric parameters of height and weight were measured by a Seca 220 stadiometer and a Seca 711 scale. Body mass index (BMI) was calculated as weight measured in kg and height indicated by m2. Smoking habit was evaluated by asking the question: “Are you currently a smoker?”.

### 2.3. Dietary Assessment

Diet and eating habits were evaluated with a validated food frequency questionnaire (FFQ) used in previous studies [36]. This selfadministered questionnaire was checked by a registered dietitian during an interview at the study center. It investigated the frequency intake of a predefined portion over the last year but not the differences in portion sizes. Each portion weight was expressed in grams. Originally, the FFQ was structured into 11 sections, representing foods with similar characteristics: grains, meat, fish, milk and dairy products, vegetables, legumes, fruits, miscellaneous foods, water and alcoholic beverages, olive oil and other edible fats, coffee/sugar, and salt. Then it was validated against the dietary records and adapted to our population [37]. In the final FFQ version, 85 food items were identified as typical local foods that reflect the local diet (Figure S1). These food items, together with some questions about the use of edible fats, have been regrouped into 28 food groups for statistical analyses [35]. The food group “edible cooking fats” could not be quantified and was not used in the present study.

### 2.4. Visual Assessment

Each participant underwent a complete ophthalmic examination (described in detail elsewhere) [17]. Briefly, the examination included best-corrected visual acuity (BCVA) measurement, slit-lamp biomicroscopy, intraocular pressure (IOP) measurement, and funduscopy. Then, we used the Optovue RTVue XR 100 AVANTI, made by Optovue, Inc., to perform OCT and OCT-A. After detecting and segmenting several retinal layers using the AngioVue module of the Optovue RTVue AVANTI program, OCT-A analyses of the retinal vasculature (version 2015.100.0.35, Optovue, Inc., Fremont, CA, USA) were performed. Both the Angio Disc mode (4.54.5 mm^2^) and the Angio Retina mode (33 mm^2^) were used. The vessel density (VD, %) was defined as the proportion of vessel area with blood flow over the total area automatically measured by the OCT software. The OCT angiograms centered on the fovea automatically defined the superficial and deep vascular plexus. The VD at each plexus of the RPE (the superficial VD (SVD), and deep VD (DVD)) were determined for the whole 3 mm circle area centered on the fovea (whole retina) (Figure S2). The thickness (µm) of the ganglion cell complex (GCC), consisting of the thickness of the retinal nerve fiber layer (RNFL), ganglion cell layer (GCL), and inner plexiform layer (IPL) (Figure S2) at the macular area, and, separately, of the RNFL, was measured at the same time using the same OCT. The device measures GCC and RNFL thickness within an automatically rendered 7 mm^2^ area, centered 1 mm temporally to the fovea [18]. Each retinal feature shown in Figure S2 is explained in Table S1. Ocular exclusion criteria for all study participants included an IOP > 22 mmHg, a history of glaucoma, optic neuropathies, demyelinating disorders, retinal diseases, including macular degeneration, diabetic or hypertensive retinopathy, epiretinal membrane, retinal detachment, an obvious media opacity reducing visual acuity below 1 LogMar and interfering with the OCT and OCT-A analysis, a refractive error of 6 diopters or more, an intraocular surgery performed in the previous 6 months, or ocular trauma.

### 2.5. Statistical Analysis

Subject characteristics are reported as Median and Interquartile Range (IQR) for continuous variables and as frequencies and percentages (%) for categorical variables. 

To test the nonnormal distribution of variables Kolmogorov–Smirnov test for equality was used.

To analyze the difference between two subgroups of age classes, the median value calculated on our cohort was chosen to cut the distribution of age variables perfectly in two parts.

To select the predictors of the OCT variable, random forest (RF) was used. RF was computed by an ensemble of binary decision trees, which could be used to select the most important variables linked with the outcomes. Variable predictiveness could be assessed using variable importance measures for both single and grouped variables [38]. The random forest (RF) method is a machine-supervised learning algorithm based on a randomized decisional tree for ranking the prediction power of a set of variables regarding the outcome of interest. The “forest” it builds is an ensemble of decision trees, usually trained with the “bagging” method. The general idea of the bagging method is that a combination of learning models increases the overall result. We examined which variables best predict variance in the intervention effects by ranking the covariates in order of importance. The ranking is calculated as the sum of how often a given covariate is split at each depth of the forest. The sum is weighted so that early splits (low forest depth) are more important than late splits. Variables are considered “more important” if the variable is more frequently used for the first splits across all decision trees that are grown in the random forest. The parameter used for ranking was the importance score variable, calculated by adding up the improvement in the objective function given by the splitting criterion over all internal nodes of a tree and across all trees in the forest (separately for each predictor variable). The importance score variable was normalized by dividing all scores over the maximum score (100%).

Variables with high importance were the drivers of the outcome, and their score values have a significant impact on the outcome [36].

We used another statistical methodology to evaluate variable importance; in fact, predictor importance was estimated based on the minimal depth of the maximal subtree. The “Depth” was how many nodes down in the tree, starting the numbering at 0 for the root node, while “Minimal depth” was the minimal depth value for the first instance of a given splitting variable. “Mean minimal depth” was the minima depth for a variable averaged across all trees in the forest.

If a predictor was influential in a prediction, then the variable is likely to occur nearer to the root rather than the leaf nodes [39]. Depth is indicated by a vertical bar with the mean value. The smaller the mean minimal depth, the more important the variable and the higher up the y-axis the variable will be. The color gradient reveals the min and max minimal depth for each variable. The range of the x-axis is from zero to the maximum number of trees for the feature. 

We randomly split the data into training and testing subgroups to predict visual outcomes. The training data included 75% of the sample (n = 397), while the remaining data (the test data) accounted for 25% (n = 133) and were used to test the model and minimize the heterogeneity of the obtained subsamples for a continuous outcome.

All statistical computations were made using StataCorp. 2021. Stata Statistical Software: Release 17. College Station, TX, USA: StataCorp LLC. And RStudio software (“Prairie Trillium” Release).

## 3. Results

The present study was conducted on a sample of 530 subjects, with a median (IQR) age of 72.00 (68.00–78.00) years, drawn from the “Salus in Apulia Study” population. The female sex was slightly predominant, accounting for 55.7%. Only 7.2% of subjects studied were smokers, while excess weight, expressed by median (IQR) BMI of 28.81 (25.98–32.04), was more common. Table 1 shows the sociodemographic and OCT variables of the sample.

Table 2 reports the average consumption of the food groups. There are differences in food consumption by gender and age groups. As regards gender, the difference is statistically significant for dairy (*p*: 0.005), eggs (*p*: 0.05), white meat (*p*: 0.009), red meat (*p*: <0.0001), processed meat (*p*: <0.0001), fish (*p*: 0.04), seafood/shellfish (*p*: 0.001), root vegetables (*p*: 0.005), grains (*p*: 0.02), high-calorie drinks (*p*: 0.04), ready to eat dish (*p*: 0.02), wine (*p*: <0.0001), beer wine (*p*: <0.0001), and spirits (*p*: <0.0001). As regards the age classes, the difference is statistically significant for processed meat (*p*: 0.009), nuts (*p*: 0.001), olives and vegetable oil (*p*: 0.006), high-calorie drinks (*p*: 0.006), ready-to-eat dishes (*p*: <0.0001), coffee (*p*^: <0.0001), beer (*p*: 0.003), and spirits (*p*: 0.05).

### 3.1. Random Forest Food Group and Retinal Nerve Fiber Layer (RNFL)

**Figure 1:**
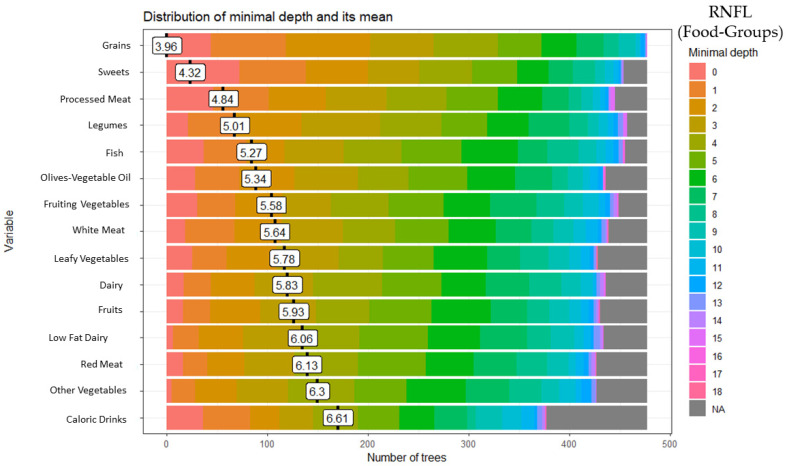
Random forest food group and retinal nerve fiber layer (RNFL).

These analyses identified 15 of the 28 food groups as predictors of RNFL: grains (minimal depth: 3.96), sweets (minimal depth: 4.32), processed meat (minimal depth: 4.84), legumes (minimal depth: 5.01), fish (minimal depth: 5.27), olives-vegetable oil (minimal depth: 5.34), fruiting vegetables (minimal depth: 5.58), white meat (minimal depth: 5.64), leafy vegetables (minimal depth: 5.78), dairy (minimal depth: 5.83), fruits (minimal depth: 5.93), low-fat dairy (minimal depth: 6.06), red meat (minimal depth: 6.13), other vegetables (minimal depth: 6.30), and high-calorie drinks (minimal depth: 6.61) (Figure 1). 

### 3.2. Random Forest Food Group and Ganglion Cell Layer (GCL) Thickness

**Figure 2:**
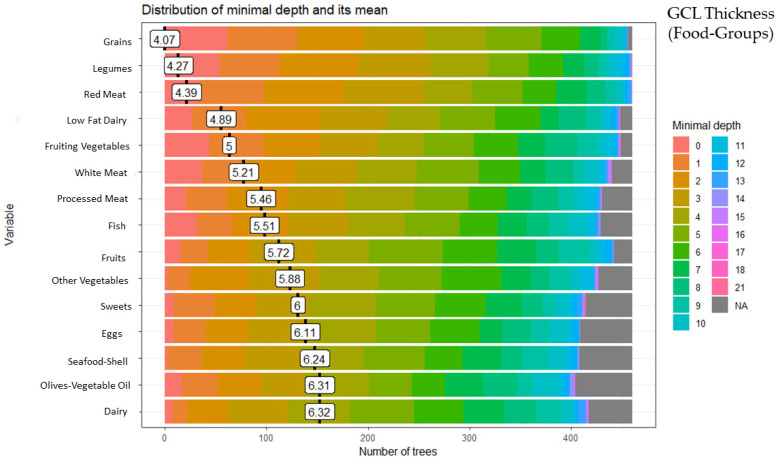
Random forest food group and ganglion cell layer (GCL) thickness.

These analyses identified 15 of the 28 food groups as predictors of GCC thickness: grains (minimal depth: 4.07), legumes (minimal depth: 4.27), red meat (minimal depth: 4.39), low-fat dairy (minimal depth: 4.89), fruiting vegetables (minimal depth: 5.00), white meat (minimal depth: 5.21), processed meat (minimal depth: 5.46), fish (minimal depth: 5.51), fruits (minimal depth: 5.72), other vegetables (minimal depth: 5.88), sweets (minimal depth: 6.00), eggs (minimal depth: 6.11), seafood-shellfish (minimal depth: 6.24), olives-vegetable oil (minimal depth: 6.31), and dairy (minimal depth: 6.32) (Figure 2). 

### 3.3. Random Forest Food Group and Whole Retina Superficial Vessel Density (SVD)

**Figure 3:**
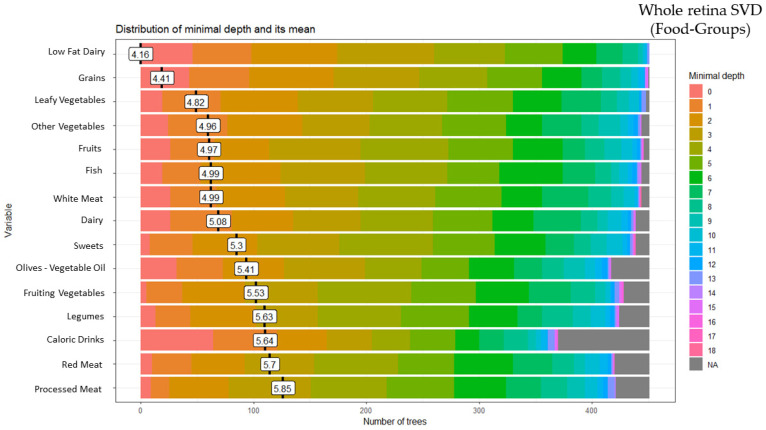
Random forest food group and whole retina superficial vessel density (SVD).

These analyses identified 15 of the 28 food groups as predictors of the whole retina SVD: low-fat dairy (minimal depth: 4.16), grains (minimal depth: 4.41), leafy vegetables (minimal depth: 4.82), other vegetables (minimal depth: 4.96), fruits (minimal depth: 4.97), fish (minimal depth: 4.99), white meat (minimal depth: 4.99), dairy (minimal depth: 5.08), sweets (minimal depth: 5.30), olives-vegetable oil (minimal depth: 5.41), fruiting vegetables (minimal depth: 5.53), legumes (minimal depth: 5.63), high-calorie drinks (minimal depth: 5.64), red meat (minimal depth: 5.70)and processed meat (minimal depth: 5.85) (Figure 3). 

### 3.4. Random Forest Food Group and Whole Retina Deep Vessel Density (DVD)

**Figure 4:**
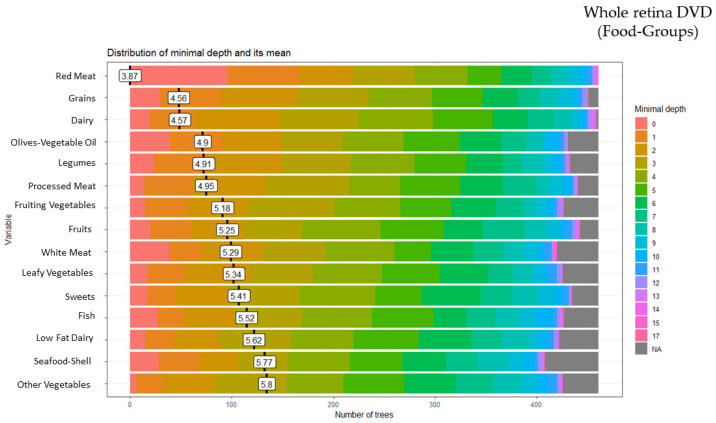
Random forest food group and whole retina deep vessel density (DVD).

These analyses identified 15 of the 28 food groups as predictors of the whole retina DVD: red meat (minimal depth: 3.87), grains (minimal depth: 4.56), dairy (minimal depth: 4.57), olives-vegetable oil (minimal depth: 4.90), legumes (minimal depth: 4.91), processed meat (minimal depth: 4.95), fruiting vegetables (minimal depth: 5.18), fruits (minimal depth: 5.25), white meat (minimal depth: 5.29), leafy vegetables (minimal depth: 5.34), sweets (minimal depth: 5.41), fish (minimal depth: 5.52), low-fat dairy (minimal depth: 5.62), seafood-shellfish (minimal depth: 5.77), and other vegetables (minimal depth: 5.80) (Figure 4). 

Table 3 summarizes the results from the random forest analyses for each variable investigated in the visual assessment. A total of 13 out of the 15 food groups were found to be common among the representative rankings of the four retinal variables selected for this study.

## 4. Discussion

The present study, which was conducted on 530 subjects aged over 64 years in Castellana Grotte, sought to identify a dietary pattern that was predictive of the variables studied, emphasizing a link between the consumption of some food groups and specific neurovascular retinal features. The foods with a high prediction power include grains, legumes, fruiting vegetables, other vegetables, fruits, olives-vegetable oil, fish, dairy, low-fat dairy, red meat, processed meat, and sweets. 

The present findings are in line with previous studies that refer to specific foods that owe their effects to the composition of micro and macronutrients. In fact, growing attention is being paid to the role of nutrition in the health of the retina and visual impairment. Several studies suggested antioxidant and anti-inflammatory effects of some specific foods [40,41]. The AREDS1 and AREDS2 pieces of research focused on specific nutritional intake and dietary supplements as strategies for preventing and slowing down AMD development. AREDS1 studied the impact of some antioxidant nutrients, including vitamin C, vitamin E, beta-carotene, and zinc, while for AREDS2, lutein, zeaxanthin, docosahexaenoic acid (DHA), eicosapentaenoic acid (EPA) were added and beta-carotene was removed [13,42]. The results were encouraging, showing a 25% reduction in the probability of developing advanced forms for subjects at high risk of AMD. There seems to be no benefit in the early stages of the disease. This different food effect on the development of AMD led to a different mechanism behind the two forms being hypothesized. Early AMD may be caused by a parainflammatory response to relatively low levels of tissue stress, while late AMD may be caused by a chronic inflammatory response to local and systemic stress [43].

### 4.1. Fruits and Vegetables

Fruits and vegetables are good sources of micronutrients (folate, potassium, magnesium, vitamins A, C, E, and K) and phytochemicals [44], which are responsible for several health benefits [45,46,47]. In fact, nowadays, increasing the consumption of fruits and vegetables is recommended due to the preventive effects of some phytochemicals against several diseases, including cardiovascular disease (CVD) [48]. In order to benefit from the protective and preventive effect of fruit and vegetables against several chronic diseases, such as CVD and different types of cancer, the World Health Organization (WHO) recommends a minimum consumption of 400 g or five portions of 80 g each of fruits, greens and/or vegetables per day [49]. In addition, fruits and vegetables, in particular leafy green vegetables, are the primary dietary sources of carotenoids. Previous studies in the scientific literature have suggested that the consumption of carotenoids, specifically lutein and zeaxanthin, tends to enhance the health of the retina, protecting against the development of retinal changes [42]. Lutein and zeaxanthin are carotenoids belonging to the family of xanthophylls. The most significant sources include kale (48.0–114.7 μg/g), parsley (64–106.5 μg/g), spinach (59.3–79.0 μg/g), lettuce (10.0–47.8 μg/g), and broccoli (7.1–33.0 μg/g) [50]. The effects of these types of carotenoids on visual performance have been the object of a wide variety of research [3]. Many of these have demonstrated a positive association between the consumption of lutein and zeaxanthin, retina health, and the prevention of some retinal disorders, such as AMD. In fact, the risk of retinal changes is significantly lower in individuals that consume more dietary lutein and zeaxanthin [51,52,53]. These results were not found for the supplementation of these nutrients due to their different bioavailability. The beneficial role is due to the presence of lutein and zeaxanthin in the center of the retina, which is needed to form the macular pigment [54]. It is essential because it reduces the penetration of harmful blue light into the retinal tissues and is responsible for the antioxidant abilities of the retina.

### 4.2. Fish

Like lutein and zeaxanthin, EPA and DHA also seem to be required for adequate retinal function [55]. They contribute to the prevention of cell apoptosis and oxidative damage, the development and maintenance of photoreceptor membranes and neurotransmitters, rhodopsin activation, and rod and cone development [56]. DHA is also the major structural component of retinal membranes, so it is essential for the development of the visual system [57]. EPA and DHA are omega-3 essential PUFAs that the human body cannot synthesize autonomously and so must obtain from a diet [58]. Changes in their levels of concentration or inadequate dietary intake can promote some retinal diseases, including AMD. Liu and colleagues reported significantly lower DHA levels in individuals with AMD than in those without [56]. The role of omega-3 fatty acids in prevention and treatment is still not completely clear. However, supplementation of EPA and DHA is known to not produce the same benefits as the dietary omega-3 fatty acids on the retina. Souied and colleagues studied the effects of DHA supplementation in the prevention and delay of the progression of exudative AMD. No significant differences were identified between the DHA and placebo groups [59]. Fish is an excellent source of EPA and DHA. In particular, there are high concentrations in dark-meat fish such as salmon, mackerel, sardines, herring, anchovies, and fish oils. Chong and colleagues showed a statistically significant association between a high intake of fish and a reduction in the development of late and intermediate AMD [60]. In the Mediterranean diet (MedDiet), regular fish consumption can ensure an adequate intake of omega-3 fatty acids. The MedDiet is also characterized by a balance between PUFA omega-6 and omega-3. This is one of the elements that makes the MedDiet the best dietary pattern in AMD risk management. Omega-6 has a proinflammatory function, while omega-3 is anti-inflammatory [55,61,62]. It has been demonstrated that an alteration in the balance of dietary omega 3- and omega 6, with a high food intake of omega 6, increases the risk of developing AMD [63]. The MedDiet has been defined as a longevity determinant thanks to the properties of its components [40,41]. Considerable scientific evidence supports the protective and preventive role of this food pattern against chronic noncommunicable diseases [64].

### 4.3. Grains

Grains are an important source of complex carbohydrates, which are then broken down into glucose by the digestive processes. Carbohydrate-source foods can be classified on the basis of their glycemic index, according to the effect on blood sugar levels over a period of two hours. Pure glucose is assigned a glycemic index (GI) value of 100. High-GI foods (>70) include white bread, potatoes, white rice, cereals, honey, and refined sugar [65]. Low-GI foods (<55) include whole fruit and vegetables, whole wheat bread, pasta, oats, bran, legumes, milk, and yogurt. Previous studies compared the effect of a low-GI diet and a high-GI diet on retinal alteration risk [66,67]. The risk was found to be higher in the high-GI diet group, probably due to inflammatory processes. However, the present study did not distinguish between whole and refined grains. Further studies are, therefore, needed to support this hypothesis.

### 4.4. Olives and Vegetable Olive Oil

Several studies have demonstrated the beneficial effects of olive oil on health status [68,69]. 

In particular, extra virgin olive oil, a key food in the MedDiet, has beneficial effects on blood pressure, glycaemic control in diabetics, endothelial functioning, oxidative stress, and lipid profiles; in addition, it reduces the susceptibility of LDL to oxidation as well as concentrations of inflammatory markers, such as C-reactive protein and interleukin-6 [70]. Its nutritional and healthy value can be attributed to the bioactive components of olive oil, including monounsaturated fatty acids (MUFAs) and PUFAs, tocopherols, and polyphenols [71]. Many epidemiological studies, including randomized controlled trials, show that the intake of olive oil improves cardiovascular health [72]. Therefore, it is possible to assume that these beneficial effects also affect the retinal microvascular system; further studies are needed to confirm this hypothesis.

### 4.5. Dairy 

The predictive power of milk and dairy towards retinal changes needs to be clarified by further studies. Some scientific studies refer to the proinflammatory effect of dairy, with others to an anti-inflammatory effect [73]. Gopinath and colleagues found an increased odds ratio for developing AMD in the case of a low intake of dairy and calcium [74]. However, these results need to be further investigated. 

### 4.6. Red and Processed Meat

Many observational studies and meta-analyses demonstrated the association between a high intake of red and/or processed meat and chronic diseases such as obesity, type 2 diabetes, CVD, and a variety of cancers. The high consumption of these foods is also associated with an increased risk of total, cardiovascular, and cancer mortality [75]. In accordance with these results, red and processed meat are predictive factors for retinal changes. Previous studies identified red and processed meat as potential risk factors for retinal diseases, such as AMD [76,77]. The mechanism underlying this hypothesis needs further exploration. Some studies focused on the high-fat content of these foods, in particular, processed meat, such as sausage or salami [78]. Furthermore, the role of other components has been studied, including heme iron, nitrites, nitrous, and advanced glycated end products (AGEs). It seems that heme iron can increase the levels of N-nitrose components, causing damage to the retina [79]. However, AGEs increase oxidative stress and inflammation and alternate normal cellular function [80].

### 4.7. Strengths and Limitations

The strengths of the present study include its large population-based sample size and the generalizability of the results to southern, older Mediterranean populations. Moreover, this study is the first to evaluate the more specific quantitative parameters of retinal vasculature when compared to previous studies, in which the morphologic parameters were evaluated using only direct fundoscopy, which could be influenced by interexaminer variability. Another strength was the identification of a dietary pattern associated with neurovascular retinal features through a strong machine-learning approach, even though it cannot explain any biological reasons for the associations detected. In fact, the dietary pattern associated with neural and vascular retinal features is to be understood as an association ranking of certain food groups with a certain outcome. However, some limitations must also be taken into account. One of these is the nature of the study, which was cross-sectional and did not allow for the clear directionality of an association to be discerned. A further limitation is the FFQ as a dietary assessment method since its memory-based nature and consequent measurement errors make it particularly difficult to analyze small diet–disease associations.

## 5. Conclusions

The aging of populations worldwide is leading to an increase in age-related pathological conditions, including retinal changes. These reduce a person’s quality of life, increasing the risk of depression, isolation, and falls. This makes it essential to focus research on new strategies that prevent this condition, slow its evolution, or mitigate its debilitating symptoms. More attention to diet and food intake should be encouraged. Starting from the data in the scientific literature on the protective role of some nutrients against retinal alterations, such as AMD, the present study analyzed the prediction power of a certain dietary pattern on retinal neurovascular features. We found a link between specific retinal variables and some food groups, such as fruiting vegetables, other vegetables, fruits, fish, olives–vegetable oil, which are sources of carotenoids and omega-3 fatty acids that are essential nutrients for the health of the retina. Thanks to its anti-inflammatory and antioxidant characteristics, the MedDiet could be a powerful tool in this sense. More specific studies on the effects of this dietary pattern on the risk of retinal alterations are warranted.

## Figures and Tables

**Table 1:** Sociodemographic and OCT variables of the sample (n = 530). The Salus in Apulia Study.

**Parameters** *	
Sex (%)	
Male	235 (44.34)
Female	295 (55.66)
Age (yrs)	72.00 (68.00–78.00)
Age Classes (%)	
65–70	204 (38.49)
70–75	131 (24.72)
75–80	102 (19.25)
80–85	63 (11.89)
85–90	28 (5.28)
>90	2 (0.38)
Education (yrs)	6.00 (5.00–10.00)
Smoking (Yes) (%)	38 (7.17)
BMI (Kg/m^2^)	28.81 (25.98–32.04)
**OCT Variables**	
RNFL Thickness (µm)	94.00 (87.00–101.00)
GCL Thickness (µm)	93.18 (87.17–98.17)
Whole retina SVD (%)	49.17 (44.44–51.41)
Whole retina DVD (%)	54.04 (50.35–56.65)

* As median and interquartile range (IQR) for continuous variables, and as frequency and percentage (%) for categorical. Abbreviations: BMI, body mass index; RNFL, retinal nerve fiber layer; GCL, ganglion cell layer; SVD, superficial vessel density; DVD, deep vessel density.

**Table 2:** Food groups average daily consumption (expressed in grams) by the sample (*n* = 530). The Salus in Apulia Study.

Parameters *		Gender			Age Classes		
	Total	Male	Female	*p* ^^^	<72 (n = 258)	≥72 (n = 272)	*p* ^^^
*Food Groups ^¥^*							
Dairy	67.65 (30.08–139.12)	56.60 (27.78–124.08)	81.37 (37.29–153.63)	0.005	75.26 (33.29–138.97)	61.19 (28.77–141.71)	0.29
Low Fat Dairy	100.00 (8.22–202.05)	100.00 (8.22–202.05)	101.31 (8.22–202.05)	0.77	100.66 (9.86–201.31)	100.00 (8.22–202.05)	0.86
Eggs	6.84 (3.42–14.82)	6.84 (3.42–14.82)	7.41 (3.42–14.82)	0.05	6.84 (3.42–14.82)	7.12 83.42–14.82)	0.46
White Meat	18.36 (9.86–31.89)	20.38 (11.18–36.00)	15.56 (9.20–26.63)	0.009	19.94 (9.86–32.05)	16.88 (9.86–31.89)	0.60
Red Meat	16.74 (9.97–27.23)	20.49 (12.33–30.14)	14.79 (8.55–24.22)	<0.0001	17.56 (10.36–28.49)	15.89 (9.70–25.18)	0.17
Processed Meat	11.09 (4.93–19.86)	13.48 (6.16–24.82)	8.77 (4.19–16.77)	<0.0001	12.41 (5.09–23.26)	9.70 (4.27–18.11)	0.009
Fish	20.38 (10.52–33.70)	21.97 (12.00–34.85)	19.51 (9.37–32.88)	0.04	22.68 (11.18–34.85)	19.45 (9.78–32.33)	0.10
Seafood/Shellfish	7.73 (2.79–14.79)	8.63 (3.70–14.79)	6.25 (2.79–11.18)	0.001	7.73 (2.79–14.79)	7.73 (2.79–11.62)	0.40
Leafy Vegetables	41.92 (22.46–72.19)	43.15 (22.74–71.23)	41.64 (21.096–73.97)	0.77	42.63 (22.46–71.23)	41.64 (21.78–74.82)	0.76
Fruiting Vegetables	71.41 (42.94–124.04)	68.83 (44.22–126.53)	72.19 (40.11–118.49)	0.62	73.73 (44.11–127.19)	66.54 (39.83–118.43)	0.26
Root Vegetables	6.57 (1.64–14.25)	3.29 (0.82–14.24)	7.12 (3.29–14.25)	0.005	6.57 (0.82–14.25)	6.57 (3.29–14.25)	0.95
Other Vegetables	55.34 (32.05–96.82)	54.08 (30.41–92.46)	57.81 (32.99–105.59)	0.44	55.07 (32.99–108.93)	56.15 (31.87–92.03)	0.43
Legumes	32.41 (21.86–47.09)	35.59 (21.86–48.53)	29.45 (21.78–45.09)	0.09	30.27 (21.86–45.86)	32.78 (21.82–47.09)	0.27
Potatoes	8.55 (3.94–15.67)	8.55 (3.94–15.67)	8.55 (3.94–15.67)	0.06	7.23 (3.94–15.67)	8.55 (3.94–15.67)	0.30
Fruits	435.98 (243.20–826.60)	413.94 (241.12–875.08)	478.70 (245.86–792.11)	0.60	429.38 (248.41–873.64)	439.62 (236.59–800.98)	0.54
Nuts	3.29 (0.49–10.68)	3.29 (0.49–10.68)	1.97 (0.49–10.68)	0.25	3.29 (0.49–10.68)	1.97 (0.49–7.12)	0.001
Grains	125.60 (74.60–186.27)	130.68 (78.00–206.92)	119.01 (71.23–170.23)	0.02	125.60 (73.45–195.96)	126.04 (74.60–177.16)	0.57
Olives and Vegetable Oil	50.25 (32.60–57.37)	51.07 (34.56–61.77)	50.25 (32.25–57.37)	0.11	47.74 (31.40–57.37)	51.07 (38.09–59.64)	0.006
Sweets	15.79 (6.90–29.14)	16.48 (6.90–29.35)	15.48 (6.93–28.49)	0.72	16.20 (7.18–30.14)	15.71 (6.74–27.56)	0.27
Sugary	8.29 (5.00–13.29)	10.00 (5.00–15.00)	7.05 (5.00–12.05)	0.17	10.00 (5.00–15.00)	7.05 (2.67–10.82)	0.11
Juices	0.00 (0.00–2.05)	0.00 (0.00–2.05)	0.00 (0.00–2.05)	0.23	0.00 (0.00–4.11)	0.00 (0.00–2.05)	0.25
High-calorie Drinks	0.00 (0.00–1.75)	0.00 (0.00–2.63)	0.00 (0.00–1.32)	0.04	0.00 (0.00–2.63)	0.00 (0.00–1.32)	0.006
Ready-to-Eat Dishes	27.12 (9.86–44.38)	32.88 (15.62–59.73)	25.48 (9.86–44.38)	0.02	32.73 (15.62–59.73)	23.01 (8.22–32.88)	<0.0001
Coffee	50.00 (30.00–60.00)	50.00 (30.00–60.00)	50.00 (30.00–60.00)	0.67	60.00 (30.00–60.00)	30.00 (30.00–60.00)	<0.0001
Wine	0.00 (0.00–150.00)	150.00 (0.00–370.00)	0.00 (0.00–100.00)	<0.0001	0.00 (0.00–150.00)	0.00 (0.00–150.00)	0.32
Beer	0.00 (0.00–0.00)	0.00 (0.00–0.00)	0.00 (0.00–0.00)	<0.0001	0.00 (0.00–0.00)	0.00 (0.00–0.00)	0.003
Spirits	0.00 (0.00–0.00)	0.00 (0.00–6.41)	0.00 (0.00–0.00)	<0.0001	0.00 (0.00–0.00)	0.00 (0.00–0.00)	0.05
Water	652.05 (456.44–978.08)	652.05 (456.44–978.08)	652.05 (456.44–978.08)	0.21	652.05 (456.44–978.08)	652.05 (456.44–978.08)	0.11

* As mean and standard deviation for continuous variables; percentage (%) for categorical variables. *^¥^* Food groups were calculated on the quantity of daily consumption (grams). ^ Wilcoxon rank sum test (Mann–Whitney).

**Table 3:** Summary of dietary pattern predictors of retinal variables.

***RNFL***	***GCl Total Thickness***	***Whole Retina SVD***	***Whole Retina DVD***
**Grains**	**Grains**	**Low Fat Dairy**	**Red Meat**
**Sweets**	**Legumes**	**Grains**	**Grains**
**Processed Meat**	**Red Meat**	Leafy Vegetables	**Dairy**
**Legumes**	**Low Fat Dairy**	**Other Vegetables**	**Olives-Vegetable Oil**
**Fish**	**Fruiting Vegetables**	**Fruits**	**Legumes**
**Olives-Vegetable Oil**	**White Meat**	**Fish**	**Processed Meat**
**Fruiting Vegetables**	**Processed Meat**	**White Meat**	**Fruiting Vegetables**
**White Meat**	**Fish**	**Dairy**	**Fruits**
Leafy Vegetables	**Fruits**	**Sweets**	**White Meat**
**Dairy**	**Other Vegetables**	**Olives-Vegetable Oil**	Leafy Vegetables
**Fruits**	**Sweets**	**Fruiting Vegetables**	**Sweets**
**Low Fat Dairy**	Eggs	**Legumes**	**Fish**
**Red Meat**	Seafood-Shellfish	Caloric Drinks	**Low-fat Dairy**
**Other Vegetables**	**Olives-Vegetable Oil**	**Red Meat**	Seafood-Shellfish
Caloric Drinks	**Dairy**	**Processed Meat**	**Other Vegetables**

*The food groups that are in common for all four retinal variables are indicated in bold.*

## Data Availability

The datasets used and/or analyzed during the current study are available from the corresponding author on reasonable request.

## Supplementary Materials

The following supporting information can be downloaded at: https://www.mdpi.com/article/10.3390/ijerph20065108/s1, Figure S1: Food Frequency Questionnaire (FFQ); Figure S2: OCT Angiography Scans and Retinal Features; Table S1: Description of the retinal features shown in Figure S2.
