# Supplementary material for: Retinal Microvasculature and Neural Changes and Dietary Patterns in an Older Population in Southern Italy
Source: Int J Environ Res Public Health. 2023 Mar 14;20(6):5108. doi: 10.3390/ijerph20065108 (PMC10049576; doi:10.3390/ijerph20065108)
Supplement: Supplementary file 1 [file ijerph-20-05108-s001.zip › Figure S2 & Table S1.pdf]

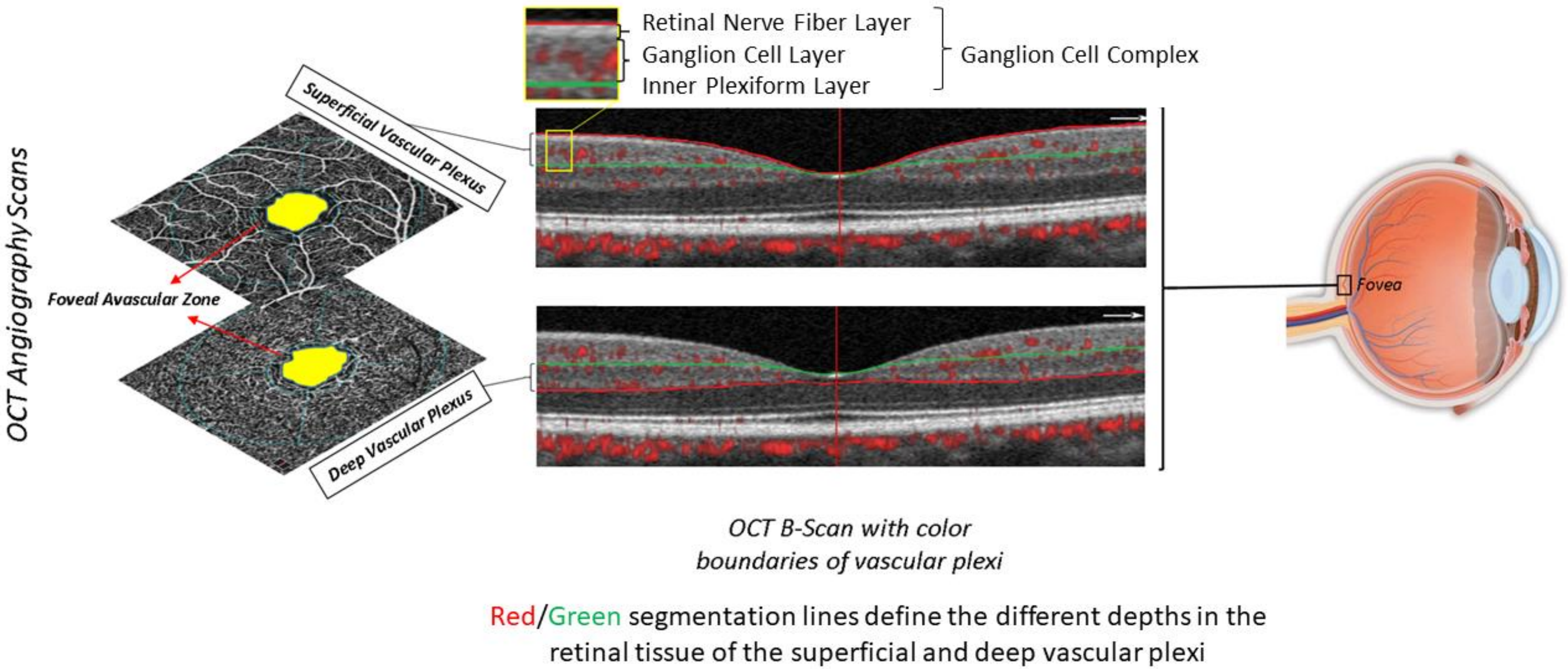

Figure S2. OCT Angiography Scans and Retinal Features

Table S1. Description of the retinal features shown in Figure S2.

		Retinal Features	Description	Legend
OCT angiography scan	Vascular plexi	Superficial vascular plexus	The superficial vascular plexus was comprised between the inner limiting membrane (red line) and 9 micron above the junction between the inner plexiform layer and the inner nuclear layer (green line). Lower vascular density indicates a poor neuroretinal vascular perfusion.	a
		Deep vascular plexus	The deep vasculare plexus was comprised between 9 micron above the inner plexiform layer junction (green line) and 9 micron below the outer plexiform layer and outer nuclear layer junction (red line).	
		Foveal avascular zone	It is a region within the fovea centralis at the center of the retina of the human eye that is devoid of retinal blood vessels. The geometric center of the FAZ is taken to be the center of the macula and thus the point of fixation.	
OCT-B scan	Ganglion cellular complex	Retinal nerve fiber layer	Is neural tissue formed by the expansion of the	b

			fibers of the optic nerve that meet the retinal cells with citoneural junctions. The thickness of the RNFL decreases with age and pathological conditions of the inner retina and optic nerve, including neurodegeneration.	
		Inner plexiform layer	This layer consists of synaptic connections between the axons of bipolar cells and dendrites of ganglion cells. To the OCT scan, the reduction of this layer is a pathological mark for the neuro-retina.	
		Ganglion cell layer	Is a neural tissue connected to the IPL and represents the inner neural layer of the retina. The reduction of GCL can be interpreted as a form of neural damage to the retina or optic nerve head, or more extensively as a neurodegeneration marker.	
